# Conversion sweet sorghum biomass to produce value-added products

**DOI:** 10.1186/s13068-022-02170-6

**Published:** 2022-06-28

**Authors:** Wei Hu, Libin Zhou, Ji-hong Chen

**Affiliations:** 1 Department of Biophysics, Institute of Modern Physics, Chinese Academy of Sciences, 509 Nanchang Road, Lanzhou, 730000 People’s Republic of China; 2 University of Chinese Academy of Sciences, Beijing, People’s Republic of China

**Keywords:** Sweet sorghum, Semi-arid agricultural land, Industrial applications, Bio-based chemicals

## Abstract

Currently, most biotechnological products are produced from sugar- or starch-containing crops via microbial conversion, but accelerating the conflict with food supply. Thus, it has become increasingly interesting for industrial biotechnology to seek alternative non-food feedstock, such as sweet sorghum. Value-added chemical production from sweet sorghum not only alleviates dependency and conflict for traditional starch feedstocks (especially corn), but also improves efficient utilization of semi‐arid agricultural land resources, especially for China. Sweet sorghum is rich in components, such as fermentable carbohydrates, insoluble lignocellulosic parts and bioactive compounds, making it more likely to produce value-added chemicals. Thus, this review highlights detailed bioconversion methods and its applications for the production of value-added products from sweet sorghum biomass. Moreover, strategies and new perspectives on improving the production economics of sweet sorghum biomass utilization are also discussed, aiming to develop a competitive sweet sorghum-based economy.

## Background

The massive population, arable land decreasing, urbanization and rising per-capita income intensify global food demand, especially for China [1–4]. For instance, the historical high of food production in China can reached 602 million metric tons in 2013 [3]. However, current industrial biotechnology in China is commonly based on bioconversion traditional sugar- or starch-containing crops to produce biochemicals (e.g., biofuels, organic acids, and other fine chemicals), but accelerating the conflict with food supply.

Many efforts have been tried to overcome the conflict and increase food supply including crop improvements [5] and the ‘Potato-as-Staple-Food’ policy by Chinese government [4], and the most competitive one is to use non-food agro-industrial feedstocks instead of traditional sugar- or starch-containing crops for industrial biotechnology utilization. Agro-industrial feedstocks including agricultural wastes, energy crops, and industrial wastes are the potential renewable non-food feedstocks for production of value-added bio-chemicals due to their abundance and low cost [3, 6–9].

Compared to other agro-industrial feedstocks, using *Sorghum bicolor* as a non-food feedstock platform, is a promising candidate for the production of value-added bio-chemicals. Based on its genotype traits and usage, so far four groups of *Sorghum bicolor* including grain, forage, fiber and sweet sorghum, has been developed and applied (Table 1).

**Table 1:** Production characteristics and usage of different varieties of sorghum

Species	Production characteristics	Usable parts	Typical/potential use	Refs.
Grain sorghum	Three to six feet tall, large ear heads	Seed	Food for humans or livestock feed	[10]
Forage sorghum	Fast-growing, high protein and fiber content	Whole plant	Feed, silage and grazing, biofuel production	[11]
Sweet sorghum	Twenty feet tall, higher biomass and lower seed yield, thicker and fleshier stems compared to grain sorghum, high content of fermentable sugars in stem	Juice or seed or bagasse or whole plant	Biofuel and biochemical production, silage for livestock feed	[8, 10, 12–14]
Fiber sorghum	Rich cellulose and hemicelluloses in stem	Stems	Paper production, biofuel and biochemical production	[15]

Especially, using sweet sorghum as a non-food feedstock, shows several following properties. First, it contains rich soluble carbohydrates, insoluble carbohydrates and bioactive compounds, and therefor can be applied to produce various desired chemicals [8, 16]. Its biomass yield can reach 60 tones/ha of stems and 2250–6000 kg/ha of seed [17], hence providing more feedstock for subsequent industry applications. Second, it has lower nitrogen fertilizer and greater water use efficiency than maize (*Zea mays* L.) [13, 18], showing lower inputs cost. Third, it can be cultivated in different environments because of its wide drought tolerance and saline–alkaline resistance. Importantly, when cultivated in margin and salinized lands, only less than 25% biomass accumulation was reduced compared with that of results in a non-saline land [19]. More specially, approximately 49.65 million ha of marginal land is available for the large-scale cultivation of sweet sorghum in China, representing the yield of 13.57 million tons sweet sorghum [20], thus providing a sustainable approaches for the efficient utilization of semi‐arid agricultural land resources.

Several valuable discussions involving in sweet sorghum biomass utilization had been conducted [7, 8, 13, 21], while these reviews mainly focused on biofuel production via microbial conversion and ensiling to ruminant feeding. This review will update recent advances of innovative technologies and its applications for value-added chemical production from sweet sorghum biomass. More specially, new perspectives on improving the production economics of sweet sorghum biomass utilization are also discussed.

### General conversion methods

**Fig. 1:**
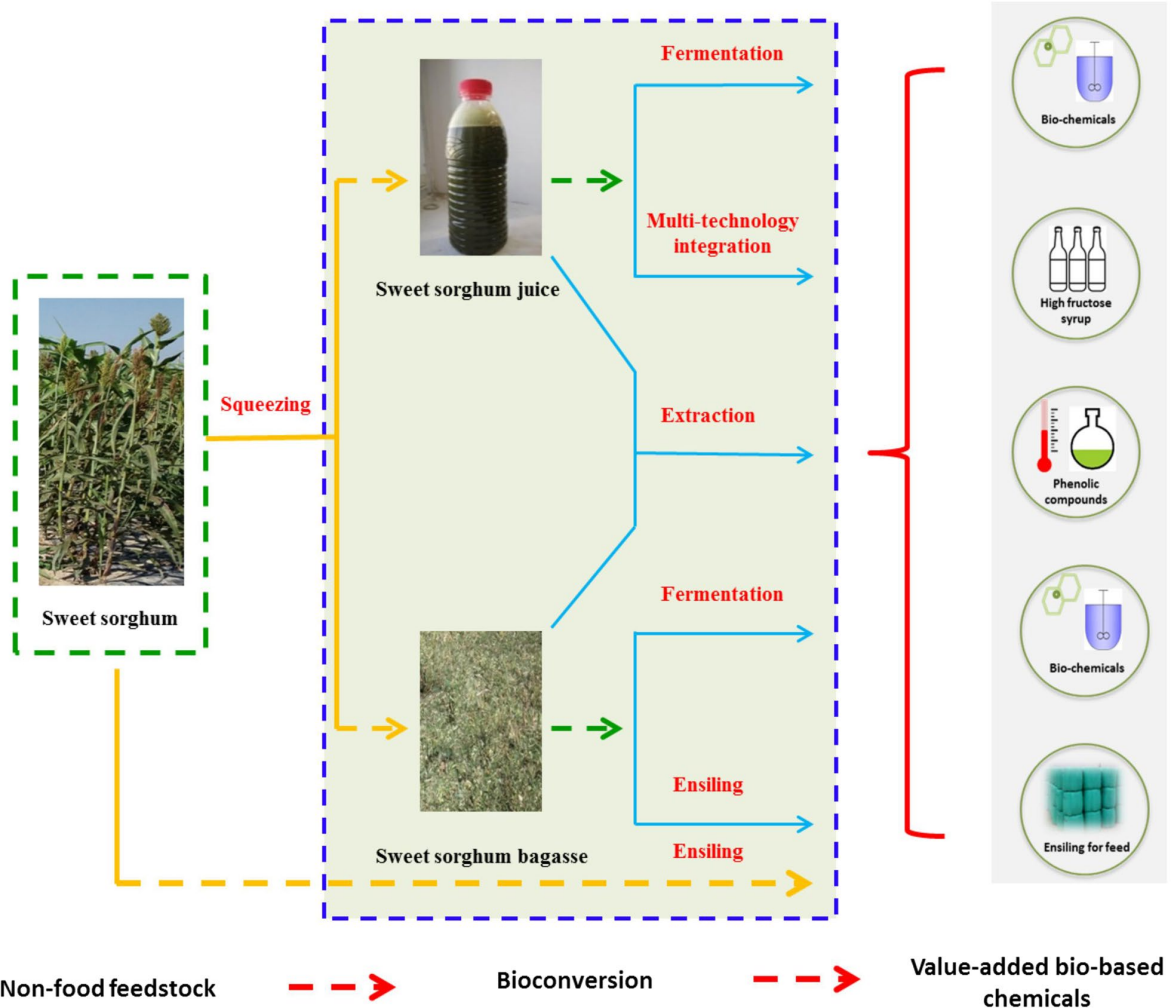
Schematic representation of value-added bio-based chemicals from sweet sorghum

As a non-food feedstock, sweet sorghum contains rich fermentable carbohydrates, insoluble lignocellulosic parts and bioactive compounds, making it more likely to produce value-added chemicals [8, 16]. Schematic representation of value-added bio-chemicals from sweet sorghum is presented in Fig. 1. Different parts of sweet sorghum have different physiological characteristics and there are also challenges when applied to downstream processes (Table 2). Thus, to solve these challenges, general conversion methods for improvement production of value-added bio-based chemicals play an important role and are summarized below.

**Table 2:** Challenges and disadvantages for downstream process using different parts of sweet sorghum as a feedstock

Usable parts	Available component	Challenges	Disadvantages	Refs.
Grain	Starch	Rich phenolic compounds	Reduced microbial productivity	[14]
Juice	Sucrose, glucose, and fructose	High-viscosity, difficult to long-term preservation, complex components	Reduced microbial productivity, easy contamination, poor dissolve oxygen	[22–24]
Bagasse	Lignin, cellulose, and hemicellulose	Complex and costly pretreatment	Generation of different inhibitors, the sugar loss	[25]

### Technological methods for bioconversion of sweet sorghum juice

Sweet sorghum juice usually are rich in soluble sugar (sucrose, glucose, and fructose), and its sugar yields vary in different sweet sorghum varieties, age of crop, climatic and edaphic factors [8]. For instance, at the pre-flowering stage, the maximum sugar concentration can reach 16–23°Brix [8]. Compared with cellulosic hydrolysates as a feedstock, bio-chemical production from sweet sorghum juice is easier and cheaper for economic production, since it does not need the costly processes of pretreatment and detoxification [22]. Using sweet sorghum juice as a feedstock, two technological methods have been conducted as follows.

### Long‐term preservation method for sweet sorghum juice

Currently, the biggest challenge for industrialization utilization of sweet sorghum juice could be attributed to the short‐term preservation period of fermentable sugars because of the contaminations by other microbes. To avoid the problem, several strategies have been tried [22, 26, 27]. Interestingly, long‐term storage of fresh sweet sorghum juice (about a 90‐day preservation at room temperature) has been developed based on the combination of evaporation and chemical preservation, showing only 5.0% fermentable sugar loss, and finally *Saccharomyces cerevisiae* M 3013 can produce 121.1 ± 4.6 g/L ethanol with a yield of 0.45 ± 0.02 g/g using the sweet sorghum juice with 90 days of preservation as a feedstock [22].

### Clarification method for sweet sorghum juice

Some impure components (such as pigment, proteins, and waxes) usually exist in crude sweet sorghum juice [28], which could influence the viscosity, and thus further lower subsequent microbial fermentation performance. Interestingly, in the study by Andrzejewski et al. [24], who developed a commercially viable hot lime clarification method to clarify sweet sorghum juice, which was beneficial to downstream microbial fermentation utilization. Using this method, Liu et al. also proved that fermentation efficiency of enduracidin yield from clarified sweet sorghum juice was improved [23].

### Technological methods for bioconversion of sweet sorghum bagasse

After juice extraction, about 50–60% wet sweet sorghum bagasse can be obtained [29], and the lignocellulosic parts generally contains 35–50% cellulose, 20–30% hemicellulose and 15–25% lignin in its bagasse [8, 30, 31], which can be also the potential feedstock for bio-chemical production via microbial conversion. To improve economic feasibility, various strategies for improving the yield of products have been developed.

### Pretreatment methods for sweet sorghum bagasse

Considering being further used by microbial cell factory, pretreatment process is essential to destroy lignocellulose recalcitrance and improve enzymatic efficiency of lignocellulosic biomass [32–34]. Several pretreatment strategies have been reported for the pretreatment of sweet sorghum bagasse [35–38], such as alkali, acid, ionic liquids, steam, acetone, microwave radiation, and fungal pretreatment. Among these methods, alkali and acid pretreatment lead to higher reducing sugar yield, but the generation of inhibitors usually hinders microbial growth and its fermentation performance, as well as corrosion issues and expensive materials for equipment construction still need to be further resolved [39].

Thus, some mild pretreatment methods are found to be the promising candidates for improving reducing sugar yield without inhibitor generation as well as better removal of lignin from sweet sorghum bagasse or sugarcane crop residue [37, 40], such as acetone pretreatment, fungal pretreatment, and inorganic salt pretreatment [41]. For example, Jafari et al. found that acetone pretreatment was a promising method for obtaining 36.3 g/L total sugar derived from enzymatic hydrolysis of sweet sorghum bagasse, leading to the production of 11.4 g/L acetone–butanol–ethanol by the bioconversion of *Clostridium acetobutylicum* NRRL B-591 [37]. After use of supplements in combination with fungal pretreatment, enzymatic hydrolysis of pretreated sweet sorghum bagasse yielded ~ 2.43 times fermentable sugar than that of untreated sweet sorghum bagasse [38]. Thus, selection of appropriate pretreatment methods will be based on the economic feasibility, operational cost and targeted bio-based chemicals [40, 41].

### Detoxification methods for sweet sorghum bagasse

In addition to the selection of appropriate pretreatment methods, a detoxification process to remove by-products from sweet sorghum bagasse including furan derivatives, organic acids and lignin derivatives, is another strategy for the consequent hydrolysis and fermentation. To detoxify inhibitors, various detoxification strategies [42–45], such as water washing, overliming, vaporization and ion exchange absorption, have been commonly used. Compared to these detoxification strategies, a detoxification method of pervaporation integrate with laccase [25], showed high detoxification efficiency of about 94.5% furfural reduction by the pervaporation method, 87.5% phenolic compounds degradation by further laccase detoxification, and low sugar loss from sweet sorghum bagasse hydrolysate. Finally, total 20.9 ± 0.12 g/L acetone–butanol–ethanol were obtained by *C. acetobutylicum* strain ABE1201 from 69.7 ± 3.2 g/L of total sugars in detoxified sweet sorghum bagasse hydrolysate.

Interestingly, a biodetoxification method using *Amorphotheca resinae* ZN1 [45], had been developed, showing excellent detoxification properties including zero energy input, zero wastewater generation, and efficient toxin degradation. Several bio-based chemicals at high concentrations has been tried, such as lysine [46], citric acid [47], lactic acid [48], cellulosic ethanol [49], gluconic and xylonic acids [50]. Thus, application of this efficient detoxification strategy will improve economic feasibility of targeted products derived from sweet sorghum bagasse, and construction of higher and lower cost detoxification technologies will still be essential in further.

### Ensiling technologies to ruminant feeding

Ensiling sweet sorghum as forage for ruminants has attracted wide attention due to its high protein content and quality of fibers [13]. In general, nutritive value of sweet sorghum silage is lower than whole corn or grain sorghum silage [51]. During feeding, fermented acidic products and residual fermentable sugar in sweet sorghum silage easily cause the growth of yeast, fungus and other pathogenic microorganisms, resulting in adverse effects on livestock [52]. To solve these problems, various ensiling technologies have been conducted. For example, silage bacteria addition is useful strategy to enhance silage quality of sweet sorghum by reducing the growth of harmful microorganisms and toxins and reducing the nutrient loss [53]. Mixing sweet sorghum with home-grown protein plant materials, is another useful strategy to improve the quality of sweet sorghum silage and provide more balanced nutrition for livestock [54–57].

### Bioactive compounds extraction methods

For high sugar crops, ethanol extraction is an effective method to extract bioactive compounds [58]. Thus, a strategy combining acidic ethanol extraction and ion precipitation was used to extract bioactive substances from sweet sorghum fresh stalks [59], and a final total phenolic concentration of 276.79 mg gallic acid equivalents (GAE)/g dried extract was obtained. Considering the safety of metal ions and improving high yield of bioactive products, a serial of environmentally friendly extraction methods need to be further tried, such as high pressure-assisted method [60], microwave-assisted method [61], ultrasound-assisted method [62], pulse electric field method [63] and supercritical carbon dioxide method [64]. For instance, a combination of acetonitrile and microwave-assisted method leaded to a high yield of flavonoid (9.69 mg catechin/g dried leaves) and phenolic compounds (10.45 mg GAE/g dried leaves) from olive tree (leaves) [65].

### Value-added products from sweet sorghum biomass

#### High fructose syrup production

As a Generally Recognized As Safe (GRAS) food sweetener, the market for high fructose syrup is huge and expanding rapidly, and starch is usually the raw material for production of high fructose syrup via enzymatic saccharification and isomerase [66]. Recently, bioconversion of inulin or beverage waste to high fructose syrup has also been tried [66, 67]. Sweet sorghum juice contains high concentration of fermentable sugars in its juice [8, 68], which is a potential feedstock for high fructose syrup production. As mentioned above, various impurities are present in sweet sorghum juice [8, 24, 28], various treatments process are essential for subsequent production of high fructose syrup. Based on that, our group has developed multi-technology integration strategy to produce high fructose syrup from sweet sorghum juice including hydrolytic process, clarification process, decoloration process, desalinization process and evaporation process, suggesting that use of non-food sweet sorghum juice is the best feedstock for producing high fructose syrup. Composition of high fructose syrup produced from sweet sorghum juice was shown in Table 3, which meets the quality standards of China National Standards (GB) GB/T 20882-2007.

**Table 3:** Composition of high fructose corn syrup produced from sweet sorghum juice

Substrate	Content
Dry substances	72%
Fructose content (assay on dry basis)	58% (w/w)
Fructose + glucose content (assay on dry basis)	97.4% (w/w)
pH	3.7
International Commission for Uniform Methods of Sugar Analysis colour	35 RBU
Insoluble matter	1.9 mg/kg
Sulfate ash	0.04%
Transparent ratio	97.9%

#### Bio-based chemical production via microbial conversion

The market for value added bio-based chemicals, such as biofuels and organic acids, is huge and increasing rapidly. For example, organic acids industry is expected to reach USD 9.29 billion by 2021 [69], and the market value of biofuel industry is about USD 153.8 billion by 2024 [63]. In this process, industrial biotechnology has emerged as a key role on the production of bio-based chemical production. Based on innovative technologies (such as clarification method, pretreatment methods, detoxification methods, and strain improvement), microbial bioprocessing technology has created bulk chemicals based on sustainable agro-industrial residues.

The rich-sugar sweet sorghum juice and insoluble lignocellulosic parts in sweet sorghum bagasse are both the potential raw materials for bio-chemical production either alone or co-fermentation mode. In addition, sweet sorghum is also used to produce some intermediate products, which are further converted to other derivatives, such as bio-butadiene [70]. Several value added industrial bio-chemicals can be produced from sweet sorghum, mainly including biofuels (Table 4) and fine chemicals (Table 5).

**Table 4:** Bio-fuels production from sweet sorghum via microbial conversion

Product	Concentration	Microorganism	Feedstock	Refs.
Ethanol	66.8–97.8 g/L	Yeast strain CAT-1	Juice	[71]
Ethanol	41.66 g/L	*Z. mobilis* R301	Juice	[72]
Ethanol	20.25% (v/v)	*S. cerevisiae*	Juice and sorghum starch	[73]
Ethanol	Yield of 88.5% and productivity of 20.3 g/L/h	Yeast strain KF-7	Juice	[74]
Ethanol	17.83 g/L	*Kluyveromyces marxianus* CCT 7735	Bagasse	[75]
Ethanol	39 g/L	*S. cerevisiae* MH1000	Bagasse	[76]
Ethanol	120.41 g/L	*S. cerevisiae* M-HT 3013	Juice and bagasse	[77]
Isopropanol and butanol	5.3 g/L isopropanol and 7.9 g/L butanol	*C. beijerinckii* DSM 6423	Mixture of sugarcane and sweet sorghum juices	[78]
Acetone–butanol–ethanol	166.5 g/L	*C. acetobutylicum* ABE 1201	Juice	[79]
Acetone–butanol–ethanol	11.4 g/L	*C. acetobutylicum NRRL B-591*	Bagasse	[37]
Acetone–butanol–ethanol	144.8 g ethanol, 17.3 g butanol and 4.8 g acetone from 1 kg sweet sorghum bagasse	*S. cerevisiae* M 3013 and *C. acetobutylicum* ABE 1201	Bagasse	[80]

**Table 5:** Value added bio-chemicals from sweet sorghum via microbial conversion

Product	Concentration	Microorganism	Feedstock	Refs.
Lactic acid	60.25 g/L	*L. Rhamnosus* LA-04-1	Juice	[81]
Lactic acid	121 g/L	*B. coagulans* and *L. rhamnosus*	Juice	[82]
Lactic acid	111 g/L	*B. coagulans* LA1507	Bagasse	[83]
Lactic acid	274.79 g/1 kg sweet sorghum stalk	*B. coagulans* LA1507	Juice and bagasse	[84]
Bio-butadiene	16 g/l kg sweet sorghum stalk	*S. cerevisiae* M 3013	Juice	[70]
Poly-β-hydroxyalkanoates	4.36 g/L	*B. aryabhattai* PKV01	Juice	[85]
	1.74 g/L	*B. aryabhattai* S4	Juice	[86]
Lysine	28.8 g/L	*Corynebacterium glutamicum* ATCC 21513	Juice	[87]
Astaxanthin	65.4 mg/L	*Phaffia rhodozyma*	Juice	[88]
	53.3 mg/L	*Phaffia rhodozyma*	Juice	[89]
Lipid	73.4% content at 50% juice concentration	*Schizochytrium limacinum* SR21	Juice	[90]
	73.26% celluar content	*Cryptococcus curvatus* ATCC 20509	Bagasse	[91]
Amylases	73.3 U/mL	*Nesterenkonia* sp. strain F	Bagasse	[92]
Co-production of hydrogen and volatile fatty acid	6.37 mmol/g-substrate H_2_, 2.33 g/L acetic acid and 2.36 g/L butyric acid	*C. thermosaccharolyticum* DSM572	Bagasse	[93]
Bacterial cellulose	2.28 g/L from root, 1.82 g/L from stalk, 2.54 g/L from leaf and 0.87 g/L from juice	*Acetobacter xylinum* ATCC 23767	Juice, root, stalk, and leaf	[94]

#### Ensiling to ruminant feeding

Food composition structure in China has shifted from mostly crops to more meat and milk due to massive population, rising per-capita income and changing dietary preferences [13, 95, 96]. Thus, increasing production of meat and milk expands the demand for livestock in the field, further accelerating demand for feed supply.

For supplying quality meats and milk, two kings of livestock feeding (such as grass feed and silage) are often conducted in developed countries [13]. However, there is a pasture shortage for several months of the year due to the climate such as South America, and thus silage for livestock feeding is the mainly style during this period of time [97]. Usually, corn is mainly cultivated for grain production and silage. In Germany, about 96% of corn was used for silage before maturity to feed cattle [13]. However, some difficulties involved in corn cultivation also continue [97, 98], such as groundwater shortages, pests and mycotoxin contamination. When compared to corn, sweet sorghum possesses some excellent properties including high tolerance to poor and acidic soils, high water-use efficiency, low nutrient requirement and high biomass production [8, 99]. Thus, ensiling sweet sorghum as a forage is a potentially candidate for ruminant feeding [13].

Recently, many efforts have been made to ensile sweet sorghum as forage for ruminants. For example, a combination ratio of sweet sorghum and alfalfa (75:25) resulted in high silage quality [54]. Similarly, based on 2% addition of oil-extracted microalgae or addition of 10% grape pomace and lactic acid bacteria, sweet sorghum silage showed improved fermentation quality, aerobic stability and feed-nutritional value [55, 56]. In another work, addition of *Lactobacillus plantarum* HY1 and *Pediococcus acidilactici* HY2 also significantly improved fermentation quality of sweet sorghum silage [53]. Ensiling sweet sorghum with Paiaguas palisadegrass also resulted in a promising silage quality with high crude protein and in vitro dry matter digestibility [100]. Ensiling sweet sorghum and korshinsk pea shrub at a ratio of 80:20 showed excellent nutrient supply for subsequent ruminant production [101].

After juice extraction, sweet sorghum bagasse can also be used as silage. For example, Dong et al. [31] added two lactic acid bacteria strain (*Lactobacillus plantarum* MN022576 and *Lactobacillus buchneri* MN022577) into sweet sorghum bagasse, showing a better silage quality including low pH value, high lactic acid accumulation and high water soluble carbohydrates. A study involving in fermentation characteristics and composition differences of sweet sorghum bagasse silages derived from different sweet sorghum varieties has also been evaluated [102]. Junior et al. also ensiling sweet sorghum bagasse had a promising fermentation characteristics and nutritive value [103].

### Utilization for phenolic compounds production

Bioactive compounds from grape seeds, *buckwheat hulls*, and *Chrysanthemum morfolium* flower extract, exhibit excellent antioxidant and radical scavenging properties, showing a promising use for food industry [62, 104, 105]. The global market value for food antioxidant reached 787 million USD in 2007 and the demand is expanding rapidly [16]. In a previous study, widespread medical and food uses of bioactive compounds in grain and sweet sorghum varieties has been reviewed [16]. Xu et al. [106] also reported that sorghum grain were rich in phenolic compounds, such as ferulic acid, gallic acid, etc. The concentration of *p*-coumaric and ferulic acids in stalk bark and pith of sweet sorghum were also measured, and stalk bark showed higher yields of *p*-coumaric and ferulic acids after alkaline hydrolysis at 170 ℃ [107]. Thus, sweet sorghum could be a cost-effective feedstock for the retrieving phenolic compounds products, and a successful case has been tried by Chen et al. [59], who developed a combined strategy to extract phenolic compounds (such as gallic, gentisic, trans-ferulic, caffeic, coumaric, chlorogenic, and phydroxybenzoic acids) from sweet sorghum fresh stalks, and extracted phenolic compounds had excellent inhibiting food-borne pathogens property.

## Future perspectives

Various bio-chemical production has been achieved from sweet sorghum biomass and efficient technologies have also been developed to improve the yield and quality of these desired products, but poor economic competitiveness is still need to be addressed. We believe that the most cost-effective way lies in further improvements of economic competitiveness of sweet sorghum biomass through at least two strategies as following:

### (1) Engineering of sweet sorghum varieties with special use and robust microbial cell factory

#### Engineering sweet sorghum varieties with special use

Considering that fermentable sugar components can be easier used to produce subsequent bulk production of value added products, it is expected that engineering of sweet sorghum varieties with different proportion and composition of fermentable sugar, will be a promising candidate for improvements of economic competitiveness of sweet sorghum biomass. In this case, identifying targeted genes/quantitative trait loci in sweet sorghum, is a crucial step, as these targeted genes/quantitative trait loci will accelerate the construction of sweet sorghum varieties with high concentration of fermentable sugars via molecular genetics tools and genome editing methods. In addition, when grown in a saline land, sweet sorghum showed 25% reduction of biomass accumulation [19], and approximately 10–20% of global arable land suffered from salinity [108]. Thus, screening target genes for the construction of sweet sorghum varieties with high salinity tolerance is also very critical. Of course, a sweet sorghum variant with combinations of these two features will even be better.

#### Engineering robust microbial cell factory

After juice extraction, lignocellulosic parts of sweet sorghum bagasse contains 37.74% cellulose, 28.07% hemicelluloses and 21.48% lignin [31]. Before being used as feedstock for microbial utilization, these components are usually treated by different pretreatment procedures to reduce intrinsic recalcitrance for improving enzymatic efficiency [109]. However, the pretreatment process results in lignin degradation, triggering the release of phenolic compounds and further hindering growth of fermentative microbial cell factories, which will increase production cost.

Besides developing efficient detoxification technology [45], construction of recombinant microbial strains with improved tolerance to inhibitors (such as screening target genes for stress tolerance, metabolic engineering, adaptive laboratory evolution, and random mutagenesis), is a promising strategy [110]. For instance, Yan, et al. [111] employed laboratory adaptive evolution to identify the gene *ZMO3_RS07160* responding to phenolic aldehydes tolerance in *Zymomonas mobilis* Z198, and overexpression of *ZMO3_RS07160* in wild type *Z. mobilis* 8b increased its phenolic aldehyde (vanillin) tolerance and cellulosic ethanol production. Expression of magnesium transporter *mgtA* in *Escherichia coli* significantly enhanced inhibitory effects of lignocellulosic hydrolysate and succinic acid production [112]. In the study of Wu et al. [113], after a multiplex atmospheric and room temperature plasma mutagenesis combined with screening, *Zymomonas mobilis* AQ8–1 and AC8–9 were generated, showing enhanced tolerance against acetic acid.

Xylose usually accounts for 30% of the total carbohydrates in cellulosic biomass [114], and construction of xylose-assimilating pathways in microbial strains is an another strategy to enhanced xylose utilization of sweet sorghum bagasse hydrolysate. Qiu et al. [114] realized the reconstruction of pentose phosphate pathway in *Pediococcus acidilactici* and xylose-assimilating ability of the constructed strain was further improved via laboratory adaptive evolution, leading to high L-lactic acid accumulation (130.8 g/L) and xylose conversion rate (94.9%) from wheat straw. Based on the metabolic engineering and adaptive laboratory evolution, *Z. mobilis* 8b-S38 was obtained [115], showing faster xylose consumption rate and the ethanol yield than that of parental strain.

Synergistic microbial community of different microbial strains is also a promising candidate strategy for enhancing yield of chemicals from sweet sorghum bagasse. For instance, a direct isobutanol production from cellulosic biomass by co-culturing of *Trichoderma reesei* esei RU and *E.coli* NV3 pSA55, was reported [116].

### (2) Extending new industrial application chain

Based on high degree of fermentable carbohydrates in sweet sorghum juice, a new way for producing desired bio-chemicals via catalytic conversion, improves its economic benefit. Some organic acids and furan chemicals has been obtained via this way [117, 118]. In the study of Bai et al. [119], 5-ethoxymethylfurfural was efficient produced via catalytic fructose conversion using a magnetic solid acid catalyst. Similarly, a continuous strategy to produce 5-hydroxymethylfurfural with high-yield from fructose (94 mol% yield) and glucose (nearly 70 mol% yield) was developed [120].

After juice extraction, about 21.48% lignin exist in sweet sorghum bagasse [31], and usually forms a cross-link recalcitrance with hemicelluloses and cellulose. Unfortunately, after destroying the recalcitrance by different pretreatment methods, phenolic compounds are generated derived from lignin degradation, further repressing microbial growth and decreasing its fermentation efficiency [121]. Thus, extraction and comprehensive utilization of lignin are an additional way for improving economic competitiveness.

After lignin extraction, residual hemicelluloses and cellulose in sweet sorghum bagasse are easier utilized via catalytic conversion or enzymatic hydrolysis for subsequent microbial fermentation. For instance, butene oligomers production from cellulose and hemicellulose was reported via catalytic production [122]. A co-production of furfural and levulinic acid from hemicellulose and cellulose has been also realized [123]. In this case, screening suitable extraction methods along with little cellulose degradation from sweet sorghum bagasse, is the crucial. Interestingly, Fu et al. developed an ionic liquid extraction method from straw, showing efficient lignin extraction and recovery, and little cellulose degradation [124]. In addition, extracted lignin from sweet sorghum bagasse is also a potentially raw material to produce other desired chemicals. For instance, a published case showed that protocatechuic acid was produced from lignin valorization by an engineered *S. cerevisiae* [125].

## Conclusions

In this review, we summarized the components of sweet sorghum, and its bioconversion methods and application for the production of value-added products. In general, bio-chemical production from sweet sorghum biomass, is of great interest owning to its low-cost and renewability. However, economic competitiveness challenges still exist. Fortunately, with the development of efficient technologies, such as synthetic biology, genome editing, catalytic conversion and lignin extraction, sweet sorghum biomass utilization and its products yield and the production economics will be further improved.

## Data Availability

We declare that all data generated or analyzed during this study are included in this article.
